# Assessment of Progression-Free Survival as a Surrogate End Point of Overall Survival in First-Line Treatment of Ovarian Cancer: A Systematic Review and Meta-analysis

**DOI:** 10.1001/jamanetworkopen.2019.18939

**Published:** 2020-01-10

**Authors:** Xavier Paoletti, Liz-Anne Lewsley, Gennaro Daniele, Adrian Cook, Nozomu Yanaihara, Anna Tinker, Gunnar Kristensen, Petronella B. Ottevanger, Gerasimos Aravantinos, Austin Miller, Ingrid A. Boere, Robert Fruscio, Anna K. L. Reyners, Eric Pujade-Lauraine, Andrea Harkin, Sandro Pignata, Tatsuo Kagimura, Stephen Welch, James Paul, Eleni Karamouza, Rosalind M. Glasspool

**Affiliations:** 1 Groupe d’investigateurs national des Etudes des Cancers Ovariens (GINECO), Paris, France; 2 Gustave Roussy Cancer Center and Institut National de la Santé et de la Recherche Medicale Oncostat, Villejuif, France; 3 Department of Biostatistics, University of Versailles St Quentin, Institut Curie, Saint-Cloud, France; 4 Scottish Gynaecological Cancer Trials Group (SGCTG), Cancer Research United Kingdom Clinical Trial Unit, Institute of Cancer Sciences, University of Glasgow, Glasgow, United Kingdom; 5 Multicenter Italian Trials in Ovarian Cancer and Gynecologic Malignancies (MITO), Clinical Trials Unit, Istituto Nazionale Tumori– Istituto di Ricovero e Cura a Carattere Scientifico (IRCCS), Fondazione G. Pascale, Napoli, Italia; 6 Medical Research Counsel Clinical Trials Unit, University College London, London, United Kingdom; 7 Japanese Gynecologic Oncology Group (JGOG), Jikei University School of Medicine, Tokyo, Japan; 8 Canadian Cancer Trials Group (CCTG), University of British Columbia, Vancouver, British Columbia, Canada; 9 Nordic Society of Gynaecological Oncology, Norwegian Radium Hospital, Oslo, Norway; 10 European Organisation for Research and Treatment of Cancer, Radboud University Medical Center, Nijmegen, the Netherlands; 11 Hellenic Cooperative Oncology Group, General Oncology Hospital of Kifissia, Nea Kifissia, Greece; 12 Gynecologic Oncology Group (GOG), Roswell Park Comprehensive Cancer Center, Buffalo, New York; 13 Department of Medical Oncology, Erasmus Medical Center Cancer Institute, Rotterdam, the Netherlands; 14 University of Milan Bicocca, San Gerardo Hospital, Monza, Italy; 15 University of Groningen, University Medical Center Groningen, Groningen, the Netherlands; 16 Association de Recherche sur les Cancers dont Gynécologiques–GINECO, Université Paris Descartes, Assistance Publique–Hôpitaux de Paris, Paris, France; 17 MITO, Istituto Nazionale Tumori di Napoli IRCCS Fondazione G Pascale, Napoli, Italy; 18 JGOG, Foundation for Biomedical Research and Innovation at Kobe, Translational Research Center for Medical Innovation, Kobe, Japan; 19 CCTG, London Health Sciences Centre, London, Ontario, Canada; 20 Gustave Roussy Cancer Center, Villejuif, France; 21 SGCTG, Beatson West of Scotland Cancer Centre, NHS (National Health Service) Greater Glasgow and Clyde, Glasgow, United Kingdom

## Abstract

**Importance:**

The Gynecologic Cancer InterGroup (GCIG) recommended that progression-free survival (PFS) can serve as a primary end point instead of overall survival (OS) in advanced ovarian cancer. Evidence is lacking for the validity of PFS as a surrogate marker of OS in the modern era of different treatment types.

**Objective:**

To evaluate whether PFS is a surrogate end point for OS in patients with advanced ovarian cancer.

**Data Sources:**

In September 2016, a comprehensive search of publications in MEDLINE was conducted for randomized clinical trials of systematic treatment in patients with newly diagnosed ovarian, fallopian tube, or primary peritoneal cancer. The GCIG groups were also queried for potentially completed but unpublished trials.

**Study Selection:**

Studies with a minimum sample size of 60 patients published since 2001 with PFS and OS rates available were eligible. Investigational treatments considered included initial, maintenance, and intensification therapy consisting of agents delivered at a higher dose and/or frequency compared with that in the control arm.

**Data Extraction and Synthesis:**

Using the meta-analytic approach on randomized clinical trials published from January 1, 2001, through September 25, 2016, correlations between PFS and OS at the individual level were estimated using the Kendall τ model; between-treatment effects on PFS and OS at the trial level were estimated using the Plackett copula bivariate (*R*^2^) model. Criteria for PFS surrogacy required *R*^2^ ≥ 0.80 at the trial level. Analysis was performed from January 7 through March 20, 2019.

**Main Outcomes and Measures:**

Overall survival and PFS based on measurement of cancer antigen 125 levels confirmed by radiological examination results or by combined GCIG criteria.

**Results:**

In this meta-analysis of 17 unique randomized trials of standard (n = 7), intensification (n = 5), and maintenance (n = 5) chemotherapies or targeted treatments with data from 11 029 unique patients (median age, 58 years [range, 18-88 years]), a high correlation was found between PFS and OS at the individual level (τ = 0.724; 95% CI, 0.717-0.732), but a low correlation was found at the trial level (*R*^2^ = 0.24; 95% CI, 0-0.59). Subgroup analyses led to similar results. In the external validation, 14 of the 16 hazard ratios for OS in the published reports fell within the 95% prediction interval from PFS.

**Conclusions and Relevance:**

This large meta-analysis of individual patient data did not establish PFS as a surrogate end point for OS in first-line treatment of advanced ovarian cancer, but the analysis was limited by the narrow range of treatment effects observed or by poststudy treatment. These results suggest that if PFS is chosen as a primary end point, OS must be measured as a secondary end point.

## Key Points

**Question:**

Is progression-free survival a validated surrogate end point for overall survival in first-line systemic treatment of ovarian cancer?

**Findings:**

In this systematic review and meta-analysis of 17 unique trials with individual data from 11 029 unique patients, a high correlation between progression-free and overall survival was found at the individual level, but a low correlation was found at the trial level.

**Meaning:**

These findings suggest that overall survival is the preferred end point in trials of first-line treatment or maintenance treatment, and progressive-free survival must be supported by additional end points if used as the primary end point.

## Teaser

This systematic review and meta-analysis evaluates whether progression-free survival is a validated surrogate end point for overall survival in patients with advanced ovarian cancer.

## Introduction

In 2012, approximately 240 000 women worldwide were diagnosed with an advanced ovarian, epithelial, fallopian tube, or primary peritoneal cancer.^1^ Approximately 75% of women have Fédération Internationale de Gynécologie et d’Obstétrique (FIGO) stage III or IV cancer at diagnosis. Initial management involves the combination of surgical cytoreduction and systemic chemotherapy. Carboplatin and paclitaxel constitute the universal standard regimen in the management of ovarian cancer, with a response rate of approximately 65%, median progression-free survival (PFS) ranging from 16 to 21 months, and median overall survival (OS) ranging from 32 to 57 months.^2^ Currently, OS is the criterion standard for the evaluation of treatment, but both OS and PFS have led to drug approvals by regulatory agencies (the US Food and Drug Administration and European Medicines Agency). Progression-free survival gives an earlier assessment of antitumor activity, requires smaller sample sizes, and is not affected by postprogression therapy. The Gynecologic Cancer InterGroup (GCIG)^3^ recommended that PFS can serve as a primary end point instead of OS, provided that secondary end points, such as quality of life, support the superiority of the investigated treatment. Evidence of the validity of PFS as a surrogate marker of OS in the modern era of different treatment types is lacking. In 2009, Buyse^4^ showed that PFS was a good surrogate marker of OS in ovarian cancer, but that study was limited to 4 trials that investigated standard cytotoxic regimens (cyclophosphamide plus cisplatin vs cyclophosphamide plus doxorubicin hydrochloride [Adriamycin] plus cisplatin) and used the older World Health Organization definition of progression. A correlation at the individual level measured by a Kendall τ of 0.84 (95% CI, 0.83-0.85) and at the group level measured by a Pearson correlation of 0.95 (95% CI, 0.82-1.00) was found. In these trials, treatment effect on PFS was associated with treatment effect on OS.

Since then, novel targeted therapies have been introduced, many of which are used as maintenance therapy. Among the tools to evaluate progression and response to treatment, cancer antigen 125 (CA125) level is an important marker in epithelial ovarian cancer.^5^ The GCIG integrated the elevation of CA125 levels into the radiological Response Evaluation Criteria in Solid Tumours (RECIST) to give a combined definition of progression.^6^ These combined criteria have never, to our knowledge, been investigated as an OS surrogate using the meta-analytic approach. Trials use different methods of assessing progression, including clinical or CA125-triggered and regular computed tomographic (CT) scans. The effect of such different assessment methods on the surrogacy of PFS also has not been assessed. To formally assess PFS measured by RECIST and combined GCIG criteria as a potential surrogate end point of overall survival, the GCIG meta-analysis group launched a prospectively planned pooled analysis of data from 11 029 individual patients (individual patient data [IPD]) and 17 randomized clinical trials of first-line therapy (initial treatment, intensification treatment, or maintenance treatment) in advanced ovarian cancers.

## Methods

This report follows the Preferred Reporting Items for Systematic Reviews and Meta-analyses (PRISMA)–IPD guidelines for the registration of the protocol, trial identification, data collection and integrity, assessment of bias, and sensitivity analyses.^7^ This meta-analysis was registered with PROSPERO (CRD42017068135). The Ethics Committee of Gustave Roussy Cancer Center, Villejuif, France, approved this study, and the French data protection authority waived the need for informed consent for the use of deidentified data.

### Trial Selection

In September 2016, a comprehensive search in MEDLINE of publications on advanced ovarian cancer was conducted. The GCIG groups were queried for potentially completed but unpublished trials. Eligible trials were randomized clinical trials of systemic treatments in patients with previously untreated ovarian cancer (or investigating maintenance treatment after first-line systemic treatment) with a minimum sample size of 60 patients in total and published from January 1, 2001, through September 25, 2016, with both OS and PFS available. Investigational treatments considered were initial, maintenance, and intensification therapy that consisted of agents delivered at higher dose and/or frequency compared with that in the control arm. The investigators of all identified trials that met the eligibility criteria were contacted for IPD sharing.

### Data and Outcomes

We requested data for all individual patients (whether or not they had been included in the primary analysis) enrolled in each trial. Overall survival was defined as the time from randomization to all-cause death or the date of the last follow-up used for censoring. Progression-free survival was defined as the time from randomization to progression or second cancer when this information was available, time to all-cause death, or the date of the last follow-up used for censoring, whichever came first. Detailed information on the type of progression was requested; this included the definition of progression, the radiological and/or clinical evaluation that documented progression, and serial measurements of CA125 levels. Assessment of progression was grouped into 3 main categories: (1) clinical examination and monitoring of 2 increases of CA125 levels to trigger CT scan confirmation of progression, (2) radiological monitoring based on RECIST, and (3) both CA125 levels and radiological assessment in line with the GCIG recommendations. Patients alive without documented disease progression were censored at the date of last follow-up. All data were centrally reanalyzed and checked for inconsistencies. In particular, diagnostic tools for randomization quality were systematically applied.^8,9^ Analysis of surrogacy was performed January 7 through March 20, 2019.

### Statistical Analysis

Forest plots were used to display the hazard ratios (HRs) overall and for individual trials, which were then used for the evaluation of surrogacy of PFS for OS. The HRs compared the hazard of an event in patients treated with an investigational regimen with the hazard in patients given the control treatment. A fixed-effect approach was implemented, and HRs were obtained from the expected and observed numbers of events. The pooled HR was then adjusted for the trial. The χ^2^ heterogeneity test and *I*^2^ statistic were used to investigate the overall heterogeneity between trials.^10^ Survival curves were estimated with the actuarial-based approach of Peto et al^11^ to account for the multiple trials. Evolution of the median survival time was assessed using a linear trend test at the trial level weighted by the number of events. Surrogacy can be evaluated at 2 different levels. At the individual level, correlation between PFS and OS means that patients with longer PFS are expected to have longer OS. However, this may only reflect the natural history of the disease, whatever the treatment is. For the assessment of the trial-level surrogacy, the treatment effect on PFS was correlated with the treatment effect on OS; in other words, we evaluated how much of the treatment effect on OS could be predicted from (or explained by) the treatment effect on PFS. We used the Kendall τ (a rank-correlation coefficient) between PFS and OS to assess surrogacy at the individual level and the coefficient of determination (corresponding to the explained variation) between the natural logarithm of the HRs for PFS and OS to assess surrogacy at the trial level.^12,13,14,15^ For both coefficients, 0 indicates absence of correlation, whereas 1.00 indicates perfect correlation. At the individual level, the association between the distribution of the true (OS) and surrogate (PFS) end points was evaluated using a bivariable model based on the Plackett copula combined with trial-specific Weibull models for PFS and OS.^13^ The treatment effects on PFS and OS were obtained from the bivariate model. The linear association between the 2 treatment effects was estimated, which in turn provided the coefficient of determination *R*^2^ for trial. Following the FLASH (Follicular Lymphoma Analysis of Surrogacy Hypothesis) initiative^16^ and a report of childhood acute lymphoblastic leukemia,^17^ a surrogate was considered to provide a reliable prediction of the treatment effect on OS from the PFS HR, when the trial-level correlation exceeded 0.8 and its 95% prediction interval excluded 0.6. This predefined threshold is arbitrary and served to limit post hoc biases (ie, choice of the threshold based on the data). Analyses were performed on an intention-to-treat basis (all patients analyzed in their allocated group irrespective of possible protocol deviations).

#### Sensitivity and Subpopulation Analysis

Leave-1-out cross-validation was implemented to assess the prediction performance of the regression model. The validation process was performed on all but 1 trial, and OS HR was predicted from the PFS HR for the left-out trial and compared with the observed value. The process was repeated for each of the 17 trials to identify potential influential trials and investigate the robustness of the results. Preplanned subgroup analyses investigated the surrogacy measures by definition of progression, by study design (initial, intensification, or maintenance treatment), and within trials that used paclitaxel and carboplatin as the control arm.

#### External Validation

To assess the external validity of our results, we used 16 trials for which we had not been able to receive IPD from the sponsors. Two of us (X.P. and E.K.) independently extracted the HRs and confidence intervals for PFS and OS from summary statistics published in these trials.^18^ The HR on PFS reported in the publication served to predict HR on OS that we in turn compared with the published HR on OS. All analyses were done using SAS, version 9.4 (SAS Institute Inc), with macros developed by Tomasz Burzykowski, PhD, and R, version X, using R surrosurv package, version 1.1.25 (R Project for Statistical Computing).^19^ Two-tailed *P* < .05 calculated using the test for heterogeneity was considered to signify statistical significance. Confidence and prediction intervals were computed at the 95% level.

## Results

### Trials’ Descriptions

**Figure 1.**
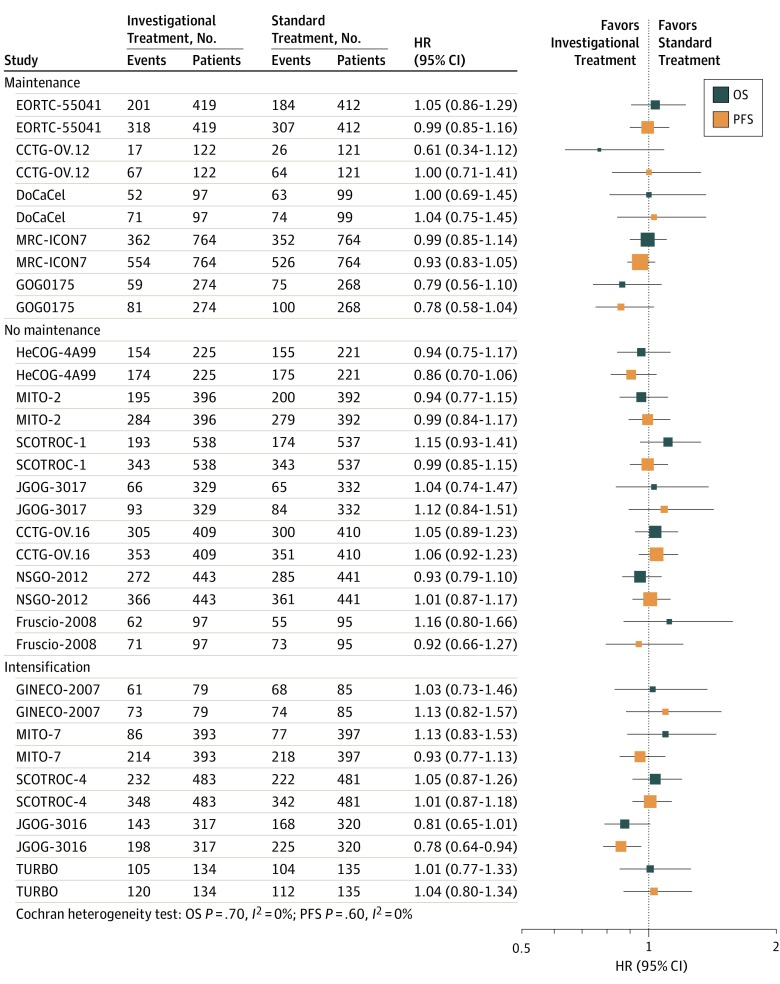
Overall and Trial by Trial Treatment Effect on Overall Survival (OS) and Progression-Free Survival (PFS) HR indicates hazard ratio. The size of the squares is proportional to the sample size of the trial.

As illustrated in eFigure 1 in the Supplement, 37 trials were identified from the literature search and their investigators were contacted. Individual patient data were obtained on 11 029 unique patients from 17 unique eligible randomized clinical trials with documented OS and PFS.^2,20,21,22,23,24,25,26,27,28,29,30,31,32,33,34,35,36^ Table 1 lists the trial-level characteristics of the 17 studies; eTable 1 in the Supplement gives an assessment of the risk of bias. In 10 trials,^20,21,24,25,26,27,28,29,32,36^ carboplatin and taxanes were the comparator. Seven studies^20,22,24,26,28,32,36^ investigated initial treatment; 5 studies,^21,24,25,30,33^ intensification treatment; and 5 studies,^23,27,31,34,35^ maintenance treatment. Four trials tested molecularly targeted treatments.^23,27,31,34^ A total of 10 trials^21,22,23,25,26,30,33,34,36^ used CA125 levels to trigger follow-up CT scans after an initial increase in the biomarker. Six trials^24,27,28,29,31,35^ used the GCIG criteria (1 multinational trial used both), and 2 trials^20,32^ used CT scan only. Data on both end points were available for all 11 029 patients, of whom 7436 experienced progression and 5138 died during follow-up. Detailed information about patients’ characteristics by allocated treatment arm and median follow-up are provided in eTable 2 in the Supplement. Median patient age was 58 years (range, 18-88 years); 5990 (54.3%) had Eastern Cooperative Oncology Group performance status of 0 at enrollment; and 8497 (77.0%) had FIGO stage III or IV disease. eFigure 2 in the Supplement shows the Peto survival curves for PFS and OS. No statistically significant time trends in the median OS and PFS according to the date of the first randomization were detected. The median OS ranged from 2.7 to 6.2 months and the median PFS ranged from 0.9 to 2.3 months (see Table 1 for median survival and eFigure 3 in the Supplement for their representation over time). No time trends according to the date of the first randomization were detected. Figure 1 shows a forest plot of the treatment effects on OS and PFS for all trials (eFigure 4 in the Supplement gives forest plots grouped by progression assessment criteria). Overall and at the trial level, the effects of investigational chemotherapy on PFS and OS were almost null (HR for PFS, 0.97 [95% CI, 0.93-1.02]; HR for OS, 0.99 [95% CI, 0.94-1.05]). No heterogeneity across trials was detected for any of the end points (*I*^2^ = 0% [*P* = .70] for OS and *I*^2^ = 0% [*P* = .60] for PFS) (Figure 1).

**Table 1.** Trial Characteristics

Source (Trial Name)	Investigational Regimen (No. of Patients)	Standard Regimen (No. of Patients)	Assessment of Progression^a^	Standard Arm, No. of Patients	Investigational Arm, No. of Patients	First Inclusion Date	Follow-up, Median (IQR), y	Median OS, y	Median PFS, y
**Maintenance**									
Vergote et al,^34^ 2014 (EORTC-55041)	Erlotinib hydrochloride (420)	Observation (415)	Clinical CA125 level (confirmation with CT)	412	419	2005	4.3 (3.8-4.8)	4.6	1.0
Hirte et al,^23^ 2006 (CCTG-OV.12)	Tanomastat (122)	Placebo (121)	Clinical CA125 level (confirmation with CT)	121	122	1998	0.9 (0.6-1.3)	NR	0.9
Reyners et al,^31^ 2012 (DoCaCel)	Docetaxel, carboplatin, and celecoxib (97)	Docetaxel and carboplatin (99)	GCIG criteria	99	97	2003	4.1 (2.6-5.7)	2.9	1.2
Oza et al,^27^ 2015 (MRC-ICON7)	Bevacizumab (764)	Standard chemotherapy (764)	GCIG criteria	764	764	2006	4.6 (4.2-5.1)	4.8	1.6
Mannel et al,^35^ 2011 (GOG-0175)	Low-dose paclitaxel (274)	Observation (268)	GCIG criteria	268	274	1998	11.6 (8.5-13.7)	NR	NR
**No Maintenance**									
Aravantinos et al,^20^ 2008 (HECOG-4A99)	Cisplatin, paclitaxel, and doxorubicin (236)	Paclitaxel and carboplatin (233)	CT scan	221	225	1999	13.7 (5.4-16.1)	3.2	1.3
Pignata et al, ^28^ 2011 (MITO-2)	Carboplatin and liposomal doxorubicin (410)	Carboplatin and paclitaxel (410)	Mixed^b^	392	396	2003	6.0 (5.0-7.1)	4.7	1.5
Vasey et al,^36^ 2004 (SCOTROC-1)	Docetaxel and carboplatin (539)	Paclitaxel and carboplatin (538)	Clinical CA125 level (confirmation with CT)	537	538	1998	2.0 (1.6-2.4)	2.9	1.2
Sugiyama et al,^32^ 2016 (JGOG-3017)	Irinotecan hydrochloride and cisplatin (332)	Carboplatin and paclitaxel (335)	CT scan	332	329	2009	3.7 (2.8-4.8)	NR	NR
Hoskins et al,^24^ 2010 (CCTG-OV.16)	Cisplatin and topotecan followed by paclitaxel and carboplatin (409)	Paclitaxel and carboplatin (410)	GCIG criteria	410	409	2002	8.2 (7.5-8.9)	3.7	1.3
Lindemann et al,^26^ 2012 (NSGO-2012)	Paclitaxel, carboplatin, and epirubicin hydrochloride (445)	Paclitaxel and carboplatin (442)	Clinical CA125 level (confirmation with CT)	441	443	1999	5.3 (4.3-5.9)	3.4	1.4
Fruscio et al,^22^ 2008	Cisplatin, ifosfamide, and paclitaxel (106)	Cisplatin, epirubicin hydrochloride, and paclitaxel (103)	Clinical CA125 level (confirmation with CT)	95	97	1997	6.8 (6.2-7.3)	4.7	1.9
**Intensification Therapy**									
Ray-Coquard et al,^30^ 2007 (GINECO-2007)	Cyclophosphamide, erubicin hydrochloride, cisplatin, and filgrastim (79)	Cyclophosphamide, erubicin hydrochloride, and cisplatin (85)	Clinical CA125 level (confirmation with CT)	85	79	1994	8.6 (6.2-9.9)	2.7	1.2
Pignata et al,^29^ 2014 (MITO-7)	Weekly carboplatin and paclitaxel (406)	Every 3 wk carboplatin and paclitaxel (404)	GCIG criteria	397	393	2008	1.9 (1.4-2.6)	4.0	1.5
Banerjee et al,^21^ 2013 (SCOTROC-4)	Carboplatin dose escalated (483)	Carboplatin flat dose (481)	Clinical CA125 level (confirmation with CT)	481	483	2005	2.7 (1.7-3.6)	2.7	1.0
Katsumata et al,^25^ 2013 (JGOG-3016)	Dose-dense carboplatin (317)	Conventional carboplatin (320)	Clinical CA125 level (confirmation with CT)	320	317	2004	6.5 (5.9-7.2)	6.2	2.3
Van der Burg et al,^33^ 2014 (TURBO)	Weekly paclitaxel and carboplatin (134)	3 Times per week paclitaxel and carboplatin (136)	Clinical CA125 level (confirmation with CT)	135	134	1998	9.4 (8.4-11.4)	3.6	1.5

Abbreviations: CA125, cancer antigen 125; CT, computed tomography; GCIG, Gynecologic Cancer InterGroup; IQR, interquartile range; NR, not reached; OS, overall survival; PFS, progression-free survival.

^a^ “GCIG criteria” indicates that patients were followed up with both serial measurements of CA125 levels and radiological measurements.

^b^ Progression of Groupe d’investigateurs national des Etudes des Cancers Ovariens (GINECO) patients was evaluated by CA125 level and confirmed by CT scan, whereas Multicenter Italian Trials in Ovarian Cancer and Gynecologic Malignancies (MITO) patients were evaluated following the GCIG guidelines.

### Individual- and Trial-Level Associations

**Figure 2.**
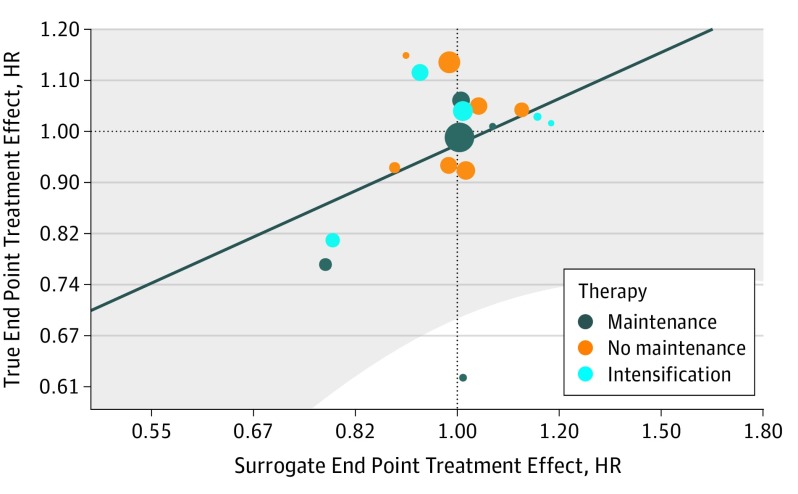
Association Between the Hazard Ratio (HR) for the Surrogate End Point Progression-Free Survival (PFS) and for the True End Point Overall Survival (OS) by Type of Trial Each trial is represented by a bubble of a size proportional to the trial sample size. The solid straight line is the linear regression model from the copula estimates that relates the PFS HR to OS HR: ln(OS HR) = 0.025 + [0.67 × ln(PFS HR)]. The shaded area corresponds to the 95% prediction limits.

The individual-level association, as measured by the Spearman rank correlation coefficient, reached 0.885 (95% CI, 0.879-0.890). The Kendall τ estimate was 0.724 (95% CI, 0.717-0.732), indicating a good correlation between PFS and OS; that is, a patient who progresses later is more likely to survive longer than a patient who progresses earlier. On the contrary, a very low correlation was noted between ln(OS HR) and ln(PFS HR) (Figure 2), where ln denotes the natural log transformation of the HR for each end point. The coefficient of determination, *R*^2^ for trial, for the estimated treatment effects was as low as 0.24 (95% CI, 0-0.59), indicating a low correlation between PFS and OS at the trial level. The linear regression model from the copula estimates was ln(OS HR) = 0.025 + [0.67 × ln(PFS HR)]. Standard errors were 0.03 and 0.31 for the intercept and slope, respectively. This is shown as a straight line in Figure 2, where the x-axis represents the treatment effect on PFS and the y-axis represents the treatment effect on OS. The shaded area corresponds to the 95% prediction limits that indicate the range of effect on OS that can be expected for a given effect on PFS, but owing to the very poor correlation, it remains largely theoretical. Despite large sample sizes, some trials with similar treatment effect on PFS had a different effect on OS, including PFS HR of greater than 1.00 together with OS HR of less than 1.00, translating into uncertainty in the prediction.

### Sensitivity Analyses

Leave-1-out cross-validation demonstrated the robustness of the results, because we had consistency between observed and predicted OS treatment effects for each trial based on the PFS (eFigure 5 in the Supplement). Only 1 strongly influential trial was identified; the OV-12 trial^23^ investigated tanomastat as maintenance therapy, which was interrupted by Bayer owing to negative results in other cancer types, and follow-up was stopped^23^; progression was assessed using CT scans after initial increase of CA125 levels. Excluding this trial increased the estimate of *R*^2^ for trial to a moderate value of 0.66 (95% CI, 0.40-0.93), more in line with previous results.

### Subgroup Analyses

Subpopulation analyses that separately focused on maintenance and nonmaintenance trials confirmed that treatment effect on PFS poorly predicted treatment effect on OS: trial-level surrogacy was low for maintenance trials (Table 2), with *R*^2^ for trial estimates from 0.03 (95% CI, 0-0.35) for maintenance vs 0.67 (95% CI, 0.36-0.97) for nonmaintenance. The marked difference was mainly explained by the OV-12 trial in the maintenance subgroup, because the *R*^2^ for the trials increased to 0.78 (95% CI, 0.40-1.00) after exclusion of this trial; the small number of trials in this subgroup strongly increased the results’ instability. Trial-level correlation was also low (*R*^2^ for trial = 0.15; 95% CI, 0-0.56) in trials that compared investigational treatment with carboplatin and taxanes. In the 6 trials^24,27,28,29,31,35^ (4603 patients) that specified GCIG guidelines to assess progression, prediction of OS HR based on PFS HR was better (*R*^2^ for trial = 0.43; 95% CI, 0.02-1.00) than that in trials that used CT scan after the initial increase of CA125 level; however, the OV-12 trial^23^ again reduced the estimated association between the treatment effects in the trials that used other assessments of progression.

**Table 2.** Overall and Subgroup Analyses of the Surrogacy of Progression-Free Survival for Overall Survival

Analysis	No. of Trials	No. of Patients	Individual-Level Correlation, Kendall τ (95% CI)^a^	Trial-Level Correlation, *R*^2^ (95% CI)^b^
Overall	17	11 029	0.724 (0.717-0.732)	0.24 (0-0.59)
Design				
Maintenance	5	3340	0.72 (0.71-0.74)	0.03 (0-0.35)
Nonmaintenance	12	7689	0.72 (0.72-0.73)	0.67 (0.36-0.97)
Carboplatin and taxanes as control	10	7321	0.73 (0.72-0.74)	0.15 (0-0.56)
Progression assessment				
CA125 level confirmed by CT scan	10	5319	0.70 (0.69-0.71)	0.27 (0-0.74)
GCIG criteria	5	4603	0.74 (0.73-0.75)	0.43 (0.02-1.00)

Abbreviations: CA125, cancer antigen 125; CT, computed tomography; GCIG, Gynecologic Cancer InterGroup.

^a^ Drawn from the joint Plackett copula model that quantifies the strength of the association between progression-free survival and overall survival for a given patient.

^b^ Indicates the determination coefficient that quantifies the strength of the association between the treatment effects on progression-free survival (progression-free survival hazard ratio) and overall survival (overall survival hazard ratio).

### External Validation

**Figure 3.**
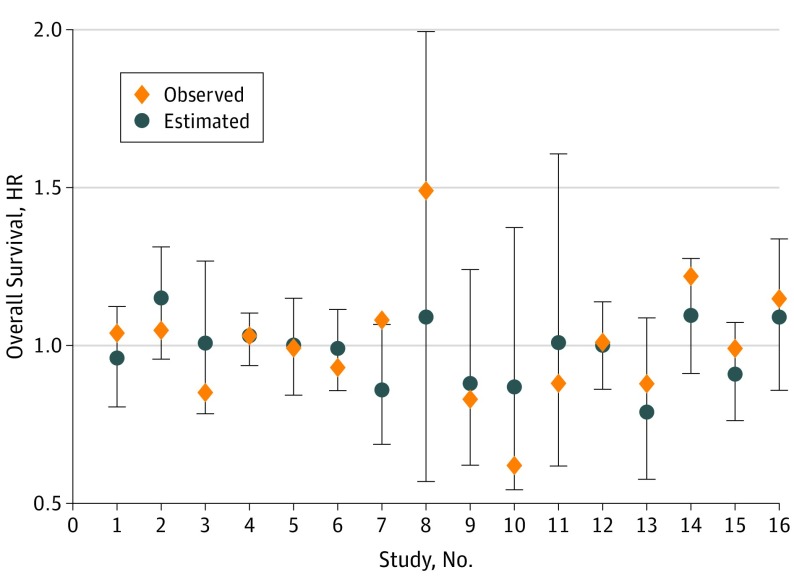
Observed and Estimated Treatment Effect on Overall Survival in Validation Trials HR indicates hazard ratio; error bars, 95% CIs.

Of the 20 trials in which we could not access the IPD (owing to refusal by the investigators,^37,38,39,40,41,42,43,44,45,46,47,48,49,50,51,52,53,54,55,56^ no response to our request, or data declared no longer available), we could extract HRs for 16 of them (8 testing initial treatments and 8 testing maintenance treatments).^37,38,39,40,41,42,43,44,45,46,47,48,49,50,51,52^ None of the trials demonstrated a statistically significant effect on OS, and only 3 studies^43,49,51^ reported a statistically significant reduction of PFS. eTable 3 in the Supplement and Figure 3 display observed OS HR and PFS HR with 95% CIs, and OS HR predicted from the model of Figure 2. Observed estimates for all except 2 trials fell within the 95% prediction intervals. However, the intervals are relatively large, reflecting the uncertainty around the prediction.

## Discussion

This pooled analysis was performed on the IPD of 11 029 patients treated in 17 randomized trials of first-line treatment for advanced ovarian cancer initiated worldwide from 1995 through 2010. Although PFS was strongly associated with OS at the individual level, we did not find a strong correlation between the treatment effects on PFS and on OS (ie, HR on PFS did not predict the HR on OS at the trial level). Low correlation was observed in maintenance and nonmaintenance therapy trials. Overall, HRs on OS and PFS were close to 1.00, with little heterogeneity among trials whether maintenance or nonmaintenance treatments were explored. All trials required rigorous response assessment schedules, with clinical and physical examination, evaluation of CA125 levels, and CT imaging. At the trial level, PFS assessed by CT scans and CA125 levels following the GCIG guidelines was moderately correlated with OS in a subgroup of 6 trials.^24,27,28,29,31,35^ Nevertheless, the role of CA125 measurements is controversial. No international standard has been established, leading to variability in calibration, assay design, and reagent specificities,^57^ and CA125 level is not considered a stand-alone marker of progression.

One trial can be seen as an outlier; the tanomastat trial was interrupted by Bayer owing to negative results in pancreatic and small cell lung cancer trials, resulting in poor follow-up for OS^23^; exclusion of this trial led to moderate trial-level associations. Nevertheless, even after exclusion of this trial, the trial-level correlation was below the predefined threshold. In trials for which we could not access IPD, no statistically significant treatment effect on OS had been reported. As external validation, we showed that for those 16 trials, the observed treatment effect (OS HR) fell within the interval predicted from PFS HR, but the interval was too large to draw accurate predictions on OS HR. These findings therefore do not support PFS as a substitute for OS in randomized clinical trials: demonstrating a reduction of PFS HR does not guarantee that a reduction in the hazard of death will be observed. If PFS is used, the GCIG criteria might be preferable as the means of assessment of progression.

Previous exploration of surrogacy in trials of first-line treatments in ovarian cancers by Buyse^4^ found high correlation, but with 4 trials that were split into subunits to increase the number of treatment effect assessments. More recently, several authors^58,59^ found moderate to high correlations at the trial level (*R*^2^ range, 0.50-0.83) from summary statistics extracted from the literature. However, unlike IPD, literature-based meta-analysis does not enable consistent calculation of end points or the full use of survival-censored data after quality checks; in addition, estimation of joint model and hence accounting for the correlated PFS and OS measured in the same patient is insufficient, leading to potential biases.

The choice of the best measure to quantify the treatment effect is controversial. Although the HR is probably the most commonly used relative measure, its validity is limited by the requirement to have a proportional hazard (ie, that the HR is constant over time). However, in the clinical trial International Collaboration on Ovarian Neoplasm (ICON7),^27^ this assumption did not hold for bevacizumab as maintenance treatment. The primary analysis was then based on an absolute measure, the difference in the restricted mean survival time between the 2 arms. The question of the surrogacy value of restricted mean survival times is to be explored in further analyses.

### Limitations

The main limit of our approach is the lack of treatment effects as measured by HR in the collected trials. Indeed, the lack of heterogeneity in the treatment effects strongly limits our ability to detect an association between PFS HR and OS HR. The regression line in Figure 2 may have been more precisely estimated if HRs had been spread across a large range. However, as shown by the validation analysis, the trials that were not collected were also negative and followed the same association between PFS HR and OS HR; additional trials should not strongly modify the conclusions obtained from this large sample. The treatment of ovarian cancers is well standardized, probably thanks to the tradition of strong collaboration within the GCIG and European Society of Gynecological Oncological Trial groups (ENGOT). Most trials enrolled large numbers of patients, were multicentric (and many international), shared the same regimen as a control, and collected similar variables. This may explain the strong homogeneity in the trials’ results; this also supports the generalizability of our findings.

A striking finding is the disappointing treatment effects measured on the PFS and the OS. This pooled analysis provides a useful benchmark for future trials. We hope that the recent improvements in PFS seen in a trial of poly–adenosine diphosphate ribose polymerase inhibitors^60^ translate into improvements in OS. So far, the combination of carboplatin and paclitaxel remains the standard chemotherapy backbone for first-line treatment.

## Conclusions

Progression-free survival cannot be validated as a strict surrogate of OS for assessing treatment effects in randomized clinical trials of first-line treatments of advanced ovarian cancers. Our findings support the GCIG Fifth Ovarian Cancer Consensus Conference statement that OS is the preferred primary end point for first-line clinical trials with or without a maintenance component,^3^ but we recognize the practical challenges and the potential for confounding factors such as crossover and long postprogression survival. Progression-free survival is an alternative primary end point, but given that we have not been able to validate it as a surrogate of OS, following the US Food and Drug Administration and European Medicines Agency guidances,^61,62^ it should represent a favorable risk-benefit association with a large magnitude of the effect or it should contribute to delaying administration of more toxic therapies as second-line treatments; therefore, if PFS is chosen, OS must be measured as a secondary end point and PFS must be supported by additional end points, such as predefined patient-reported outcomes, especially for maintenance therapy.

## Notes

Supplement. **eFigure 1.** PRISMA Flow Diagram **eFigure 2.** Overall Survival (A) and Progression Free Survival (B) **eFigure 3.** Overall Survival (OS) in Each Trial According to the Year of Trial’s Initiation **eFigure 4.** Overall and Trial by Trial Treatment Effect (HR) on Overall and Progression-Free Survival **eFigure 5.** Re-estimating the Relationship Between the Hazard Ratio (HR) on OS and HR(PFS) by Leaving One Trial Out at a Time **eTable 1.** Risk of Bias Summary: Authors’ Judgments About Each Risk of Bias Item for Each Included Study **eTable 2.** Patients’ Characteristics **eTable 3.** Observed and Predicted Treatment Effect on Overall Survival (OS HR), Based on the Observed Treatment Effect on Progression-Free Survival (PFS HR)
