# Supplementary material for: Assessment of Progression-Free Survival as a Surrogate End Point of Overall Survival in First-Line Treatment of Ovarian Cancer: A Systematic Review and Meta-analysis
Source: JAMA Netw Open. 2020 Jan 10;3(1):e1918939. doi: 10.1001/jamanetworkopen.2019.18939 (PMC6991254; doi:10.1001/jamanetworkopen.2019.18939)
Supplement: Supplement. — eFigure 1. PRISMA Flow Diagram eFigure 2. Overall Survival (A) and Progression Free Survival (B) eFigure 3. Overall Survival (OS) in Each Trial According to the Year of Trial’s Initiation eFigure 4. Overall and Trial by Trial Treatment Effect (HR) on Overall and Progression-Free Survival eFigure 5. Re-estimating the Relationship Between the Hazard Ratio (HR) on OS and HR(PFS) by Leaving One Trial Out at a Time eTable 1. Risk of Bias Summary: Authors’ Judgments About Each Risk of Bias Item for Each Included Study eTable 2. Patients’ Characteristics eTable 3. Observed and Predicted Treatment Effect on Overall Survival (OS HR), Based on the Observed Treatment Effect on Progression-Free Survival (PFS HR) [file jamanetwopen-3-e1918939-s001.pdf]

Supplementary Online Content

Paoletti X, Lewsley L-A, Daniele G, et al; Gynecological Cancer InterGroup (GCIG) Meta-analysis Committee. Assessment of progression-free survival as a surrogate end point of overall survival in first-line treatment of ovarian cancer: a systematic review and meta-analysis. *JAMA Netw Open.* 2020;3(1):e1918939. doi:10.1001/jamanetworkopen.2019.18939

eFigure 1. PRISMA Flow Diagram

eFigure 2. Overall Survival (A) and Progression Free Survival (B)

eFigure 3. Overall Survival (OS) in Each Trial According to the Year of Trial's Initiation

eFigure 4. Overall and Trial by Trial Treatment Effect (HR) on Overall and Progression-Free Survival

eFigure 5. Re-estimating the Relationship Between the Hazard Ratio (HR) on OS and HR(PFS) by Leaving One Trial Out at a Time

eTable 1. Risk of Bias Summary: Authors' Judgments About Each Risk of Bias Item for Each Included Study

eTable 2. Patients' Characteristics

eTable 3. Observed and Predicted Treatment Effect on Overall Survival (OS HR), Based on the Observed Treatment Effect on Progression-Free Survival (PFS HR)

This supplementary material has been provided by the authors to give readers additional information about their work.

© 2020 Paoletti X et al. *JAMA Network Open.*

eFigure 1. PRISMA Flow Diagram

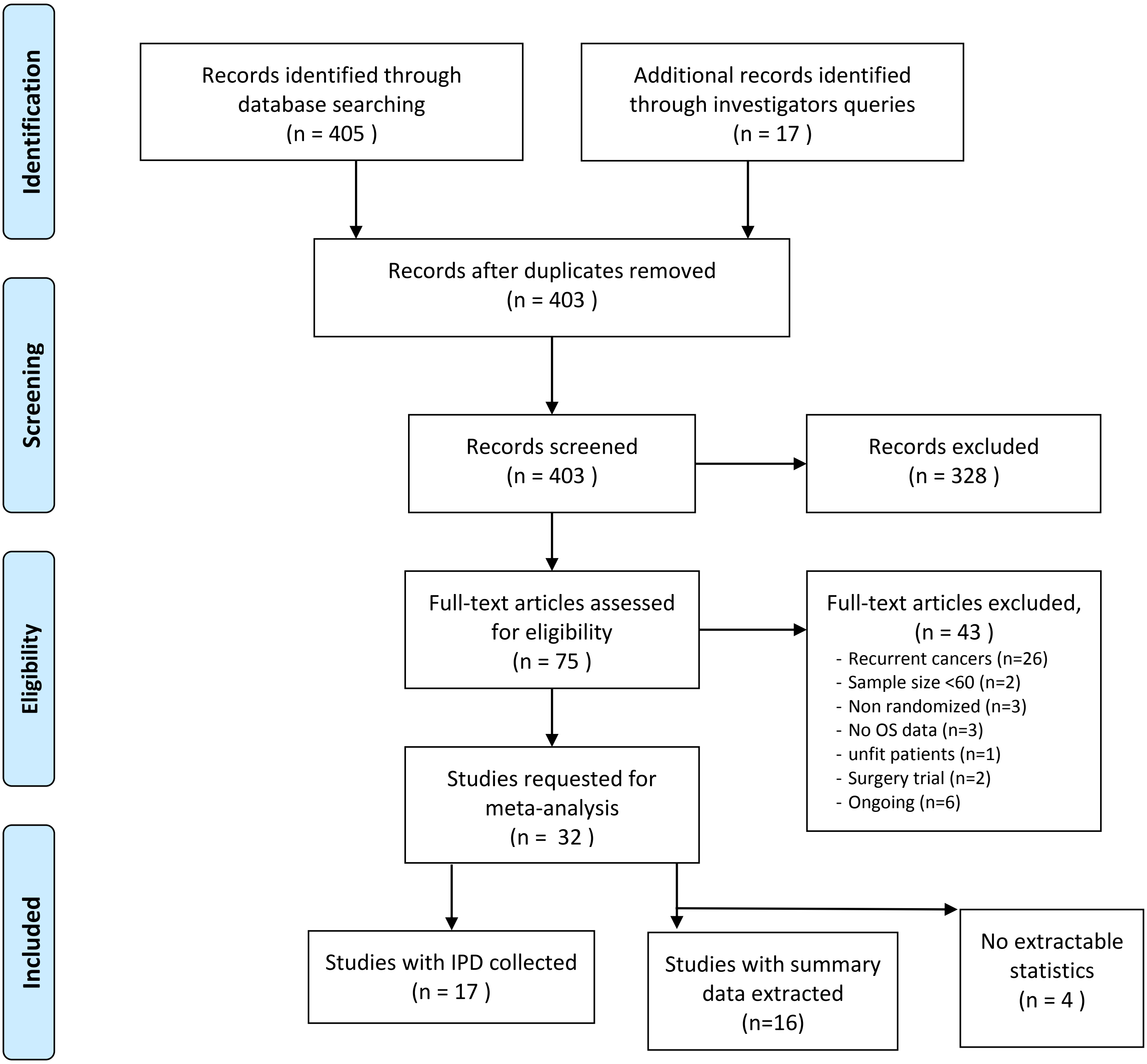

© 2020 Paoletti X et al. *JAMA Network Open.*

eFigure 2. Overall Survival (A) and Progression Free Survival (B)

- Peto Curve truncated at 8 years. Person-years are provided, which may be inferior to the number of patients at risk due to patients having event or being censored during the considered year.

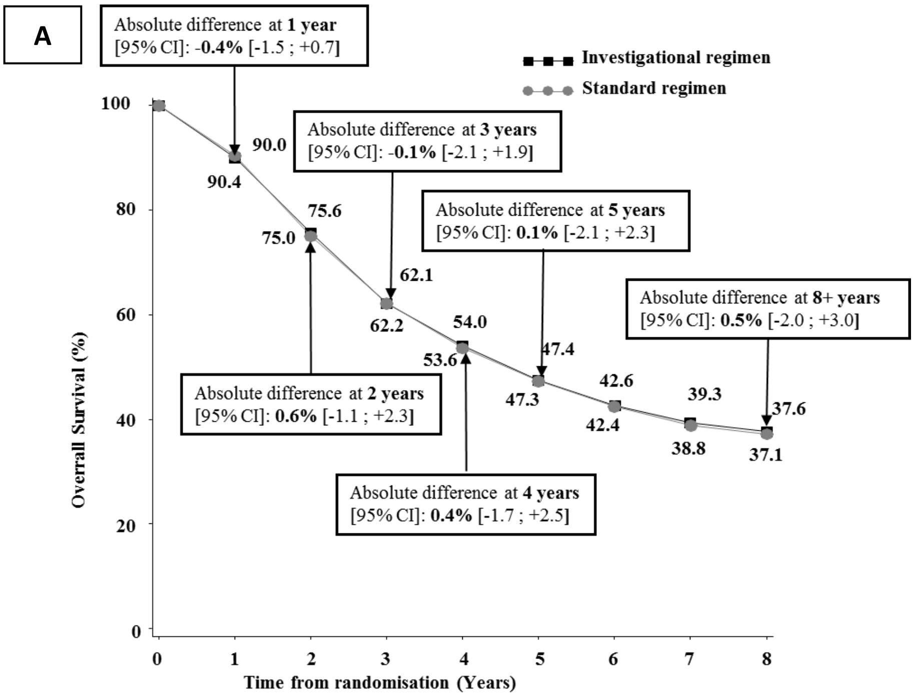

Number of deaths / person-years

Years	[0;1[	[1;2[	[2;3[	[3;4[	[4;5[	[5;6[	[6;7[	[8+[
Investigational regimen	551/5215	718/4154	586/2982	306/2184	194/1491	96/902	49/602	65/1525
Standard regimen	524/5213	779/4146	549/2940	324/2158	181/1472	97/883	53/589	66/1425

© 2020 Paoletti X et al. *JAMA Network Open.*

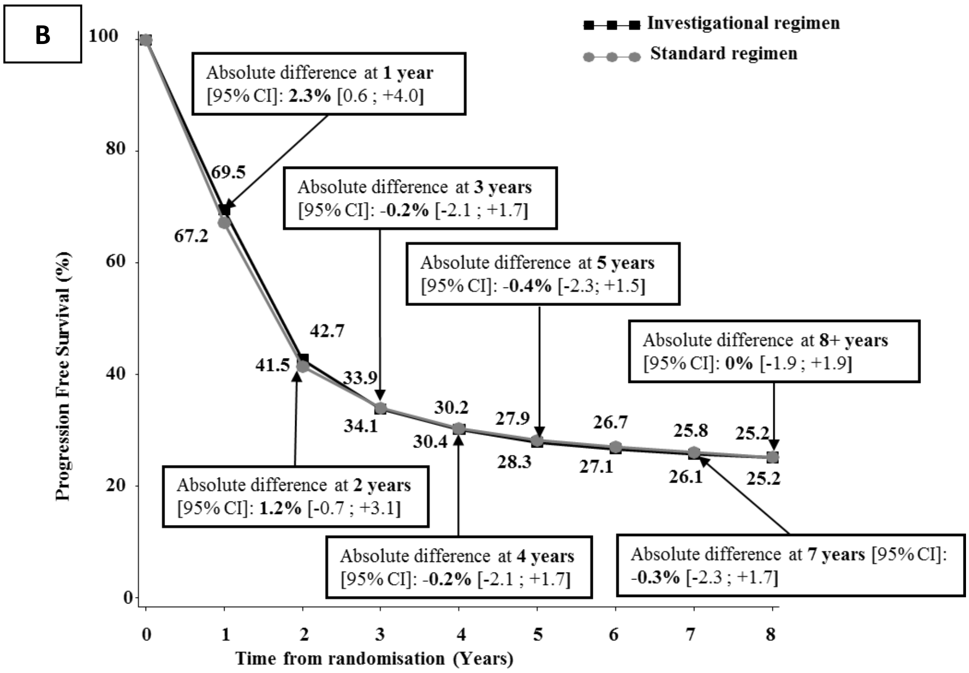

Number of progression events / person-years

Years	[0;1[	[1;2[	[2;3[	[3;4[	[4;5[	[5;6[	[6;7[	[8+[
Investigational regimen	1725/4762	1335/2731	388/1676	143/1215	66/866	26/577	13/408	32/1240
Standard regimen	1868/4682	1244/2587	319/1641	139/1211	61/867	24/555	16/396	37/1124

© 2020 Paoletti X et al. *JAMA Network Open.*

eFigure 3. Overall Survival (OS) in Each Trial According to the Year of Trial’s Initiation

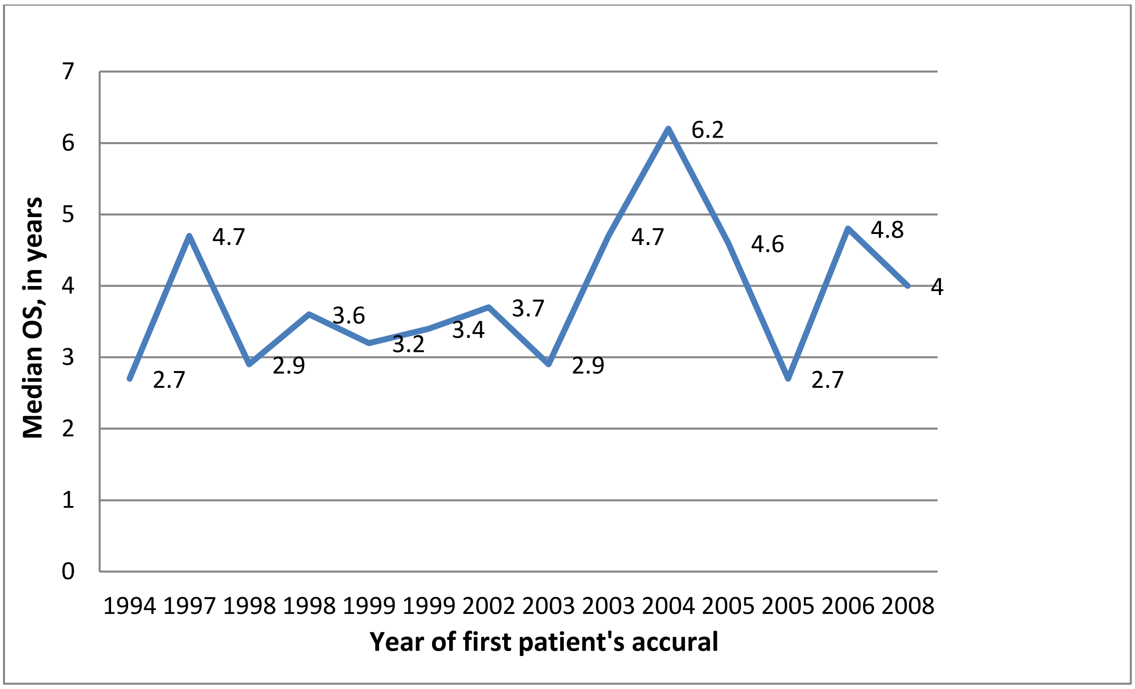

© 2020 Paoletti X et al. *JAMA Network Open.*

eFigure 4. Overall and Trial by Trial Treatment Effect (HR) on Overall and Progression-Free Survival

A, Overall and trial by trial treatment effect (HR) on overall Survival

	Events / Patients Inv. rx	Events / Patients Std rx	Statistics (O−E)	Statistics Var.	HR (95% CI)
**Assessment of progression : CT−scan only**					
HeCOG−4A99	154 / 225	155 / 221	−4.9	77.1	0.94 (0.75 ; 1.17)
JGOG−3017	66 / 329	65 / 332	1.4	32.7	1.04 (0.74 ; 1.47)
**Subtotal**	**220 / 554 (39.7 %)**	**220 / 553 (39.8 %)**	**−3.5**	**109.8**	**0.97 (0.80 ; 1.17)**
Heterogeneity Chi−square= 0.25, df= 1: p= 0.6					
**Assessment of progression : CA−125 (conf. with CT)**					
GINECO−2007	61 / 79	68 / 85	1.1	32	1.03 (0.73 ; 1.46)
EORTC−55041	201 / 419	184 / 412	5.1	96.1	1.05 (0.86 ; 1.29)
MITO−7	3 / 32	4 / 28	−1.2	1.7	0.50 (0.11 ; 2.26)
SCOTROC−1	193 / 538	174 / 537	12.5	91.6	1.15 (0.93 ; 1.41)
SCOTROC−4	232 / 483	222 / 481	5.1	113.3	1.05 (0.87 ; 1.26)
JGOG−3016	143 / 317	168 / 320	−16.7	77.6	0.81 (0.65 ; 1.01)
TURBO	105 / 134	104 / 135	0.6	52.1	1.01 (0.77 ; 1.33)
CCTG−OV.12	17 / 122	26 / 121	−5.2	10.7	0.61 (0.34 ; 1.12)
NSGO−2012	272 / 443	285 / 441	−9.6	139	0.93 (0.79 ; 1.10)
Fruscio−2008	62 / 97	55 / 95	4.2	29.2	1.16 (0.80 ; 1.66)
**Subtotal**	**1289 / 2664 (48.4 %)**	**1290 / 2655 (48.6 %)**	**−4.1**	**643.5**	**0.99 (0.92 ; 1.07)**
Heterogeneity Chi−square= 10.44, df= 9: p= 0.3					
**Assessment of progression : Both (GCIG)**					
MITO−2	195 / 396	200 / 392	−5.7	98.7	0.94 (0.77 ; 1.15)
MITO−7	83 / 361	73 / 369	6.2	39	1.17 (0.86 ; 1.60)
CCTG−OV.16	305 / 409	300 / 410	7	151	1.05 (0.89 ; 1.23)
DoCaCel	52 / 97	63 / 99	0.1	28.3	1.00 (0.69 ; 1.45)
MRC−ICON7	362 / 764	352 / 764	−2.5	178.2	0.99 (0.85 ; 1.14)
GOG0175	59 / 274	75 / 268	−8.1	33.5	0.79 (0.56 ; 1.10)
**Subtotal**	**1056 / 2301 (45.9 %)**	**1063 / 2302 (46.2 %)**	**−3**	**528.7**	**0.99 (0.91 ; 1.08)**
Heterogeneity Chi−square= 3.61, df= 5: p= 0.6					
**Total**	**2565 / 5519 (46.5 %)**	**2573 / 5510 (46.7 %)**	**−10.6**	**1281.9**	**0.99 (0.94 ; 1.05)**

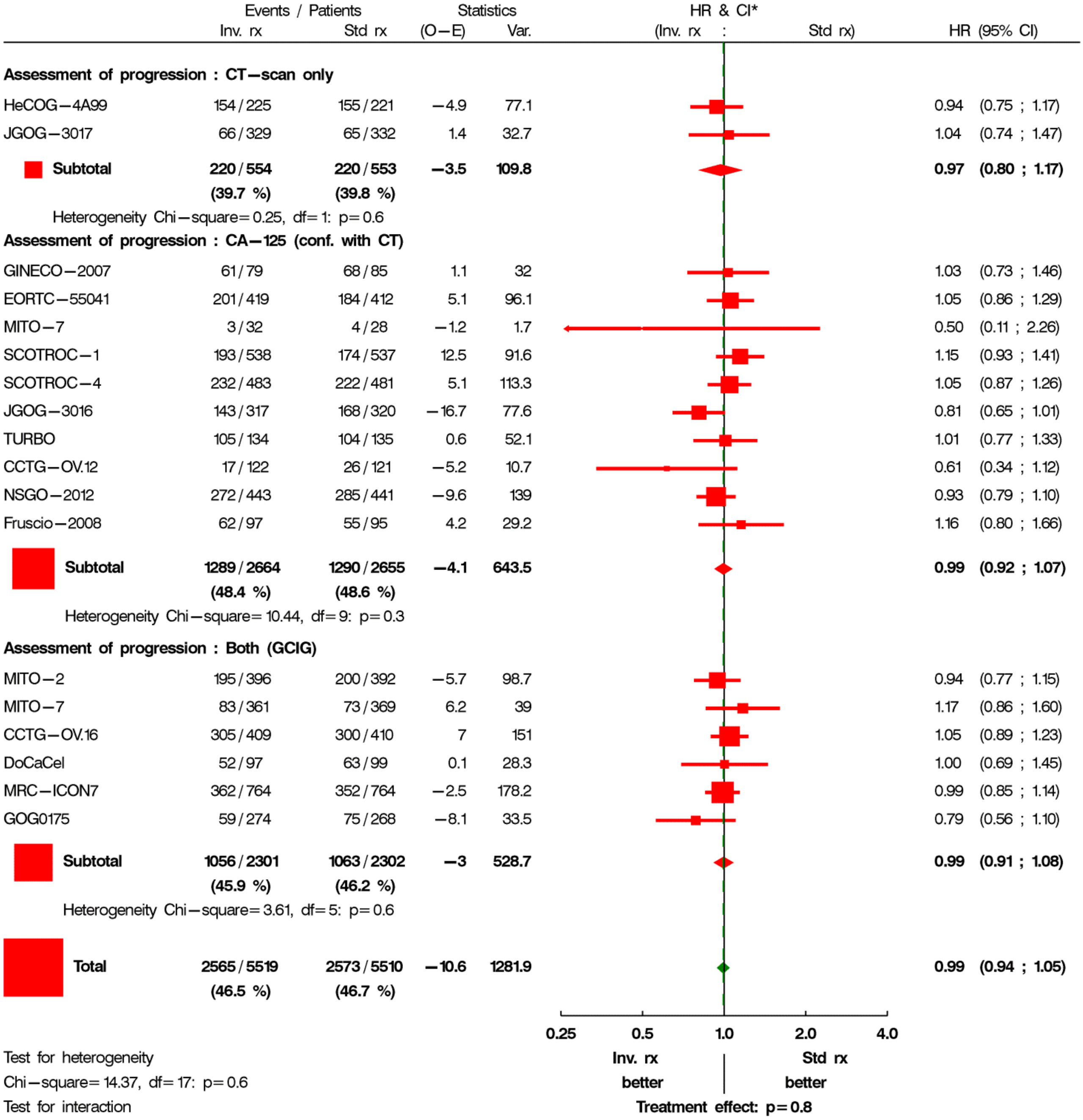

Test for heterogeneity
Chi−square= 14.37, df= 17: p= 0.6
Test for interaction
Chi−square= 0.07, df= 2: p= 1

© 2020 Paoletti X et al. *JAMA Network Open.*

B, Overall and trial by trial treatment effect (HR) on progression free survival

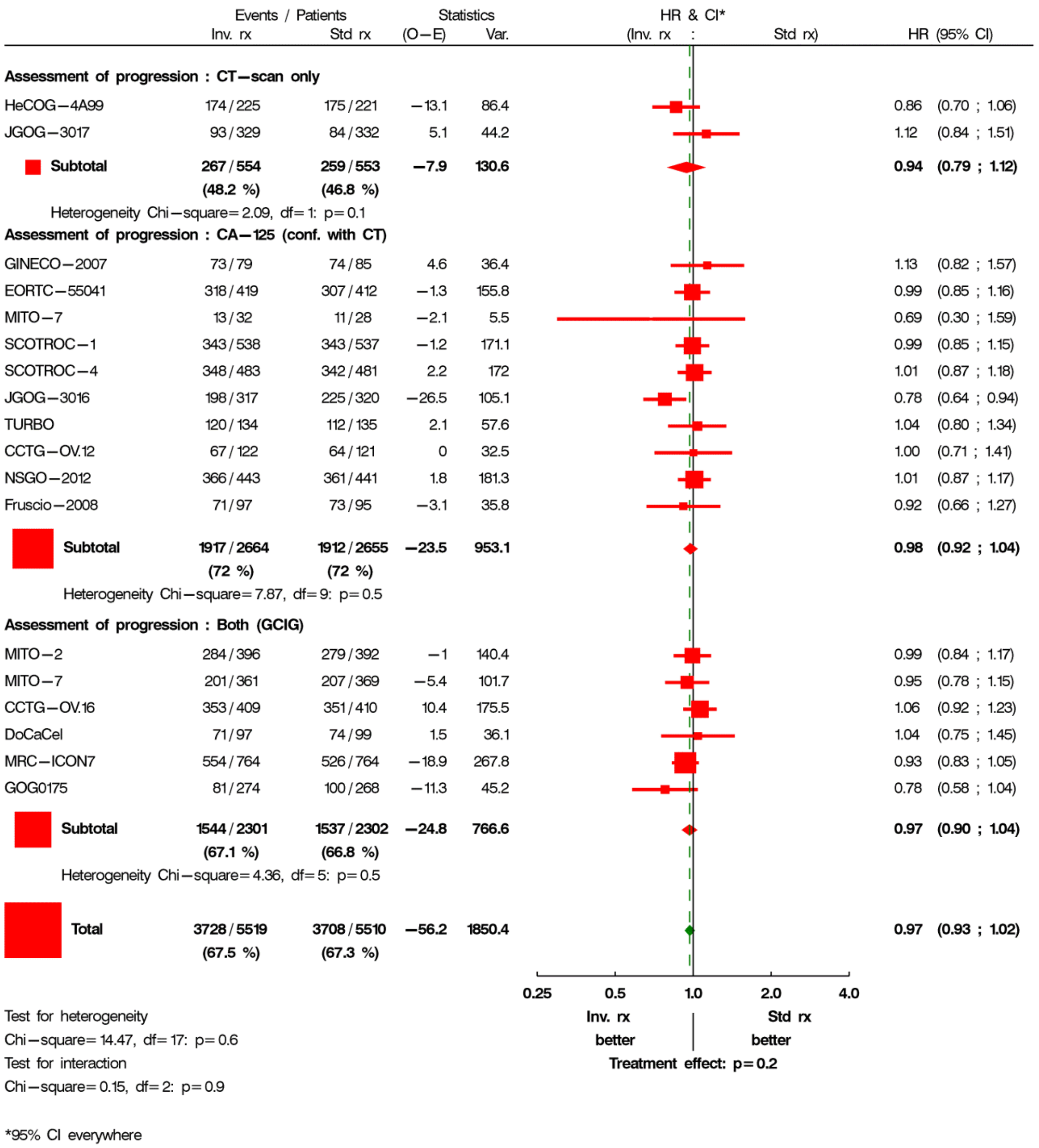

MITO-7 appears twice as part of the patients had systematic CT-scans as per the GCIG criteria while others had CT-scans in the case of CA125 biomarker raise.

© 2020 Paoletti X et al. *JAMA Network Open.*

eFigure 5. Re-estimating the Relationship Between the Hazard Ratio (HR) on OS and HR(PFS) by Leaving One Trial Out at a Time

For each labeled trial, the full Clayton copula model was estimated by excluding the labeled trial. The squares and circles indicate the HR(OS) predicted from the HR(PFS) and the observed HR(OS) estimates. The solid bars are the 95% confidence intervals (CIs). The red circle corresponds to the OV-12 trial (tanomastat as maintenance therapy that was interrupted by Bayer due to negative results in pancreatic and NSCL cancers) was the only observation outside of the predicted interval. Exclusion of the OV-12 trial gave a better correlation (R^2=0.66, 95%CI=0.40, 0.93), yet insufficient to validate surrogacy.

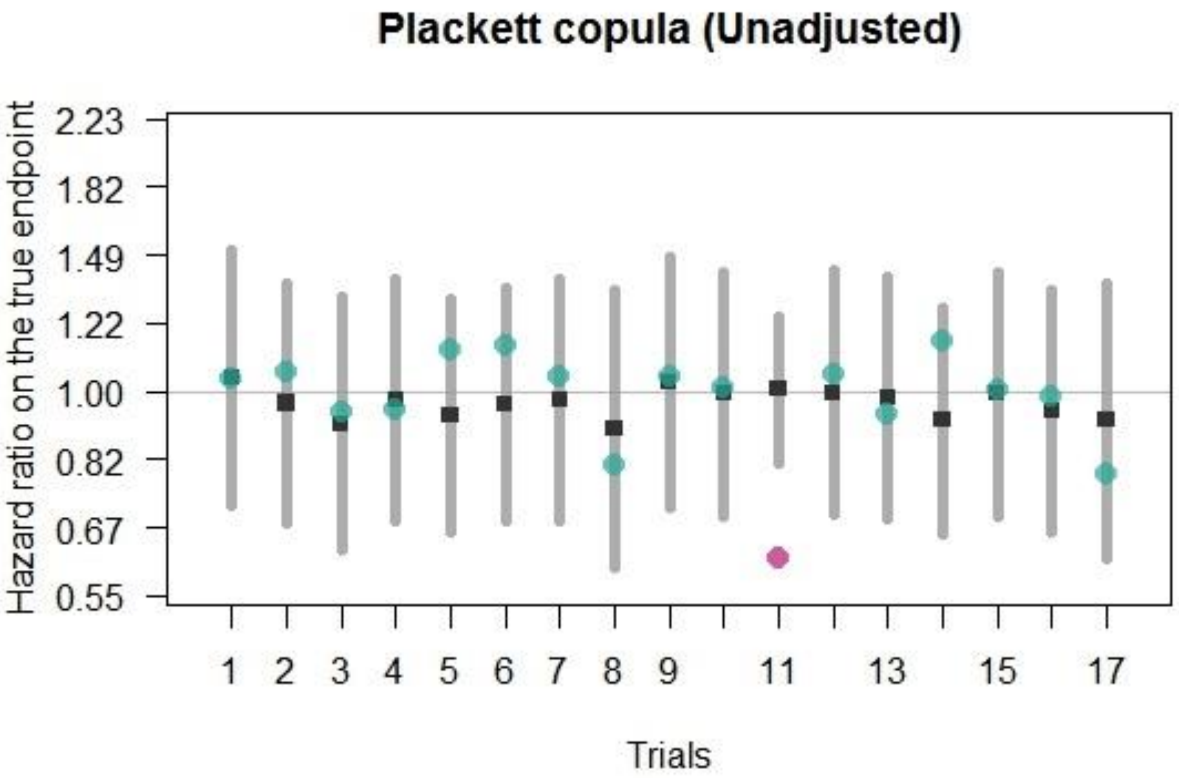

Excl. Trial	1	2	3	4	5	6	7	8	9	10	11	12	13	14	15	16	17
R^2_{copula}	0.2	0.23	0.23	0.23	0.27	0.23	0.23	0.14	0.22	0.18	0.66	0.23	0.24	0.26	0.23	0.24	0.18

Excl. stands for excluded.

© 2020 Paoletti X et al. *JAMA Network Open.*

eTable 1. Risk of Bias Summary: Authors' Judgments About Each Risk of Bias Item for Each Included Study

Items	Random sequence generation (selection bias)	Allocation concealment (selection bias)	Follow-up quality (Kaplan-Meier inversed)	Blinding of participants and personnel (performance bias)	Blinding of outcome assessment (detection bias) for OS / PFS	Incomplete outcome data (attrition bias)	Other bias
EORTC-55041	-	-	-	+	-/+	-	
CCTG-OV.12	-	-	-	-	-/+	-	
DoCaCel	-	?	-	+	-/+	-	
MRC-ICON7	-	-	-	+	-/+	-	
GOG-0175	-	-	-	+	-/+	-	
HECOG-4A99	-	-	-	+	-/+	-	
MITO-2	-	-	-	-	-/-	-	
SCOTROC-1	-	-	-	+	-/+	-	
JGOG-3017	-	-	-	+	-/+	-	
CCTG-OV.16	-	-	-	+	-/+	-	
NSGO-2012	-	-	-	+	-/+	-	
Fruscio-2008	-	?	-	+	-/+	-	
GINECO-2007	-	-	-	+	-/+	-	
MITO-7	-	-	-	+	-/+	-	
SCOTROC-4	-	-	-	+	-/+	-	
JGOG-3016	-	-	-	+	-/+	-	
TURBO	-	-	-	+	-/+	-	

Each domain was judged as 'low risk of bias' (-), 'high risk of bias' (+), or 'unclear risk of bias' (?) in each study according to the Cochrane Handbook for Systematic Reviews of Interventions (Higgins JPT, Green S. Cochrane Handbook for Systematic Reviews of Interventions. The Cochrane Collaboration; 2011)

© 2020 Paoletti X et al. *JAMA Network Open.*

eTable 2. Patients' Characteristics

Variable		Total		Standard regimen		Investigational regimen	
Age	n (m.d.)	11027		5509		5518 (1)	
	Mean (sd)	57.8 (10.8)		57.9 (10.8)		57.7 (10.9)	
	Median (Q1 ; Q3)	58 (50 ; 66)		58 (50 ; 66)		58 (50 ; 66)	
	Min ; Max	18 ; 88		18 ; 87		19 ; 88	
Performance status	n (m.d.)	10964		5480		5484	
	0	5990	54.6%	2994	54.6%	2996	54.6%
	1	4305	39.3%	2168	39.6%	2137	39.0%
	2+	629	5.7%	296	5.4%	333	6.1%
	3	40	0.4%	22	0.4%	18	0.3%
Figo	n (m.d.)	11013		5500		5513 (6)	
	Stage I(a&b)	297	2.7%	144	2.6%	153	2.8%
	Stage Ic	1049	9.5%	533	9.7%	516	9.4%
	Stage II(a&b)	669	6.1%	331	6.0%	338	6.1%
	Stage IIc	501	4.6%	242	4.4%	259	4.7%
	Stage III(a&b&c)	6689	60.7%	3366	61.2%	3323	60.3%
	Stage IV(a&b)	1808	16.4%	884	16.0%	924	16.7%

© 2020 Paoletti X et al. *JAMA Network Open.*

Histological subtype	n (m.d.)	10892 (137)		5436 (74)		5456 (63)	
	Serous carcinoma	6076	55.8%	3041	55.9%	3035	55.6%
	Endometrioid	1050	9.6%	516	9.5%	534	9.8%
	Mucinous	308	2.8%	146	2.7%	162	3.0%
	Clear cell	1358	12.5%	676	12.4%	682	12.5%
	Other	1513	13.9%	778	14.3%	735	13.5%
	Undifferentiated	127	1.2%	63	1.2%	64	1.2%
	Unknown	460	4.2%	216	4.0%	244	4.5%
Histological grade	n (m.d.)	10644 (385)		5314 (196)		5330 (189)	
	Well differentiated	842	7.9%	432	8.1%	410	7.7%
	Moderate differentiated	2206	20.7%	1091	20.5%	1115	20.9%
	Poorly/Undifferentiated	5951	55.9%	2989	56.2%	2962	55.6%
	Unknown	1645	15.5%	802	15.1%	843	15.8%
Surgical procedure	n (m.d.)	8609 (2420)		4304 (1206)		4305 (1214)	
	Primary resection	7567	87.2%	3794	88.2%	3773	87.6%
	Delayed surgery	343	4.0%	159	3.7%	184	4.3%
	No surgery	699	8.1%	351	8.1%	348	8.1%
Maximal residual disease	n (m.d.)	9085 (1944)		4540 (970)		4545 (974)	
	<1cm	5154	56.7%	2571	56.7%	2583	56.8%
	>=1cm	3931	46.3%	1969	43.3%	1962	43.2%

© 2020 Paoletti X et al. *JAMA Network Open.*

Assessment of progression	n	11029		5510		5519	
	CT-scan	1107	10.0%	553	10.0%	554	10.0%
	CA-125-Clinical(confirmation with CT)	5319	48.2%	2655	48.2%	2664	48.3%
	Both	4603	41.7%	2302	41.8%	2301	41.7%

© 2020 Paoletti X et al. *JAMA Network Open.*

eTable 3. Observed and Predicted Treatment Effect on Overall Survival (OS HR), Based on the Observed Treatment Effect on Progression-Free Survival (PFS HR)

Trial label	Validation Trials (references)	Initial (I) vs maintenance (M)	Observed PFS HR (95% CI)	Observed OS HR (95% CI)	Predicted OS HR (95% PI)
1	GOG-0218 [37]	I	0.91 (0.8, 1.04)	1.04 (0.83, 1.3)	0.96 (0.81, 1.12)
2	Gineco-TCG/OVAR-9 [38]	I	1.18 (1.06, 1.32)	1.05 (0.91, 1.19)	1.15 (0.96, 1.31)
3	Bolis-2010 [39]	I	0.98 (0.71, 1.36)	0.85 (0.56, 1.29)	1.01 (0.78, 1.27)
4	GOG0182-ICON5 [40]	I	1.01 (0.89, 1.14)	1.03 (0.94, 1.14)	1.03 (0.94, 1.10)
5	Valspodar-2008 [41]	I	0.96 (0.8, 1.15)	0.99 (0.83,1.19)	1 (0.84, 1.15)
6	AGO-OVAR-5 [42]	I	0.95 (0.83, 1.07)	0.93 (0.81, 1.08)	0.99 (0.86, 1.11)
7	AGO-OVAR16 [43]	M	0.77 (0.64, 0.91)	1.08 (0.87, 1.33)	0.86 (0.69, 1.07)
8	Herzog-2013 [44]	M	1.09 (0.72, 1.63)	1.49 (0.69, 3.23)	1.09 (0.62, 1.24)
9	Vergote-2013 [45]	M	0.80 (0.5, 1.29)	0.83 (0.52, 1.35)	0.88 (0.62, 1.24)
10	AGO-OVAR-15 [46]	M	0.78 (0.5, 1.22)	0.62 (0.36, 1.06)	0.87 (0.54, 1.37)
11	After-6 Protocol 1 [47]	M	0.98 (0.67, 1.44)	0.88 (0.50, 1.55)	1.01 (0.62, 1.61)
12	AGO- GINECO TEC-TC [48]	I	0.97 (0.85, 1.1)	1.01 (0.99, 1.52)	1 (0.86, 1.14)
13	SWOG-9701/GOG-178 [49]	M	0.68 (0.52, 0.88)	0.88 (0.75, 1.32)	0.79 (0.58, 1.09)
14	Gordon-2011 [50]	M	1.11 (0.94, 1.31)	1.22 (0.99, 1.52)	1.1 (0.1, 1.28)
15	AGO-OVAR-12 [51]	I	0.84 (0.72, 0.98)	0.99 (0.83, 1.17)	0.91 (0.76, 1.07)
16	MIMOSA [52]	M	1.1 (0.92, 1.32)	1.15 (0.72, 1.52)	1.09 (0.86, 1.34)

HR= hazard ratio; PFS = progression free survival; OS= overall survival; CI = confidence interval; PI = prediction interval

© 2020 Paoletti X et al. *JAMA Network Open.*
